# Effectiveness of screening preschool children for amblyopia: a systematic review

**DOI:** 10.1186/1471-2415-9-3

**Published:** 2009-07-16

**Authors:** Christine Schmucker, Robert Grosselfinger, Rob Riemsma, Gerd Antes, Stefan Lange, Wolf Lagrèze, Jos Kleijnen

**Affiliations:** 1 Department of Medical Biometry and Statistics (German Cochrane Center), Institute of Medical Biometry and Medical Informatics, University Medical Center, 79104 Freiburg, Germany; 2 Department of Non-drug Interventions, Institute for Quality and Efficiency in Health Care, 51105 Cologne, Germany; 3 Kleijnen Systematic Reviews Ltd, York YO26 6RB, UK; 4 University Eye Hospital, University Medical Center, 79106 Freiburg, Germany; 5 The School for Public Health and Primary Care (Caphri), Maastricht University, 6200 MD Maastricht, Netherlands

## Abstract

**Background:**

Amblyopia and amblyogenic factors like strabismus and refractive errors are the most common vision disorders in children. Although different studies suggest that preschool vision screening is associated with a reduced prevalence rate of amblyopia, the value of these programmes is the subject of a continuing scientific and health policy discussion. Therefore, this systematic review focuses on the question of whether screening for amblyopia in children up to the age of six years leads to better vision outcomes.

**Methods:**

Ten bibliographic databases were searched for randomised controlled trials, non-randomised controlled trials and cohort studies with no limitations to a specific year of publication and language. The searches were supplemented by handsearching the bibliographies of included studies and reviews to identify articles not captured through our main search strategy.

**Results:**

Five studies met the inclusion criteria. Of these, three studies suggested that screening is associated with an absolute reduction in the prevalence of amblyopia between 0.9% and 1.6% (relative reduction: between 45% and 62%). However, the studies showed weaknesses, limiting the validity and reliability of their findings. The main limitation was that studies with significant results considered only a proportion of the originally recruited children in their analysis. On the other hand, retrospective sample size calculation indicated that the power based on the cohort size was not sufficient to detect small changes between the groups. Outcome parameters such as quality of life or adverse effects of screening have not been adequately investigated in the literature currently available.

**Conclusion:**

Population based preschool vision screening programmes cannot be sufficiently assessed by the literature currently available. However, it is most likely that the present systematic review contains the most detailed description of the main limitations in current available literature evaluating these programmes. Therefore, future research work should be guided by the findings of this publication.

## Background

Amblyopia is a reversible visual deficit that develops during the maturation of the visual system (which is usually considered to be up to seven years of age) and may affect one or both eyes [1-3]. Risk factors associated with amblyopia are strabismus (a misalignment of the eyes) and uncorrected refractive errors, in particular anisometropia (unequal refractive errors between the two eyes) [4,5]. Congenital cataract, congenital ptosis and corneal injury or dystrophy can also cause amblyopia but are less common [6]. In Western countries, the prevalence rate of amblyopia among preschool children ranges between 2% and 5%, depending on the threshold value of visual acuity at a particular age [7-10]. In a multicenter study, it was shown that anisometropia was the cause of amblyopia in nearly 40% of children aged from three to under seven years. Strabismus was seen in 38% and a combination of anisometropia and strabismus was the etiology in 24% of preschool children treated for amblyopia [11].

Amblyopia is the leading cause of monocular vision loss in people aged between 20 and 70 years [12]. The lifetime risk of bilateral visual impairment in people with amblyopia aged 55 years or over is nearly doubled by the presence of this visual deficit (18%) [13]. The projected risk of vision loss affecting the non-amblyopic eye in individuals in the UK was also investigated by Rahi et al 2002, but they reported a much lower lifetime risk of bilateral visual impairment (1.2%) [14]. Moreover, amblyopia may also harm school performance and later adult self-image [15,16].

Preschool screening programmes for amblyopia were developed in response to experimental data in animals which suggested that early treatment of conditions analogous to human amblyopia is more effective than treatment later in life [17]. In some countries – for example in Sweden and Israel – these programmes have been well established [18,19]. However, countries like USA, Canada, Belgium and Switzerland have no standardised preschool vision screening programmes [19]. Although different (cross-sectional) studies, in particular from Scandinavian countries [20] and a newly released study from Israel [18], suggest that preschool vision screening is associated with a reduced prevalence rate of amblyopia, the value of these programmes is subject of a continuing scientific and health policy discussion [21]. For example, a British review from the year 1997 has suggested that preschool vision screening should be discontinued, on the grounds that there is insufficient evidence to justify it [22]. In contrast, the U.S. Preventive Services Task Force (USPSTF) recommends screening to detect amblyopia, strabismus and defects in visual acuity in children between three and four years of age [23]. Others, however, have argued that additional research is needed to ascertain the utility of preschool vision screening programmes [24].

In view of these discrepancies, we conducted an assessment according to criteria of the UK National Screening Committee [25] to determine the effectiveness of a preschool vision screening programme.

## Methods

### Systematic literature search

We searched Medline (Ovid), Embase, CINAHL, PSYCHinfo, Cochrane Central (CDSR, DARE, NHS EED, HTA), PSYNDEXplus, Social SciSearch, GIN and Medion from inception until December 2007. The search strategy was based on combinations of medical subject headings (MeSH) and keywords and was not restricted to specific languages or years of publication. The search strategy used in Medline (Ovid) is presented in Table 1. Search strategies for other databases were modified to meet the requirements of each database. However, the search algorithm was similar. Although not the focus of this review, the literature search also included terms associated with organic eye disorders, diagnostic measurements and treatment of amblyopia. The results of these searches will be presented in separate systematic reviews. The searches were supplemented by handsearching the bibliographies of included studies and reviews. Additionally, enquiries were sent to manufactures of screening instruments.

**Table 1:** Search strategy in Medline (Ovid)

	**Search term**	**Hits**
1	exp CHILD/	1129108

2	exp INFANT/	692510

3	(baby or babies or newborn or neonat$).mp.	536032

4	exp schools/	49523

5	exp CHILD-HEALTH-SERVICES/	14438

6	exp CHILD-DAY-CARE-CENTERS/	3371

7	(CHILD$ or ADOLESC$ or JUVENILE$ or MINOR$ or SCHOOL$ or KINDER-GARTEN$ or PRE?SCHOOL$ or NURSER$).ti.	529317

8	(CHILD$ or ADOLESC$ or JUVENILE$ or MINOR$ or SCHOOL$ or KINDER-GARTEN$ or PRE?SCHOOL$ or NURSER$).ab.	640000

9	1 or 2 or 3 or 4 or 5 or 6 or 7 or 8	2031820

10	exp strabismus/	9863

11	exp amblyopia/	4081

12	exp REFRACTIVE-ERRORS/	17635

13	((EYE$ or SIGHT$ or VI-SION$ or VISUAL$) adj4 (PROBLEM$ or DEFECT$ or IMPAIR$ or DEFICI$ or REDUC$)).mp. [mp = ti, ot, ab, nm, hw]	24836

14	(LAZY adj EYE$).mp. [mp = ti, ot, ab, nm, hw]	14

15	(AMBLYOPI$ or SQUINT$ or STRABISM$ or ANISO-METROPI$ or MYOPI$ or HYPERMETROPI$ or ASTIGMATI$ or AM-METROPI$ or HYPER-OPI$).mp. [mp = ti, ot, ab, nm, hw]	32219

16	cataract$.mp.	40130

17	microtropia.mp.	75

18	glaucoma.mp.	36328

19	retinoblastoma.mp.	14353

20	((heredit$ or retinal or macular) and dystroph$).mp.	5741

21	refract$.mp. [mp = ti, ot, ab, nm, hw]	77398

22	exp vision, low/	936

23	(SPECTACLES or GLASSES).mp.	4495

24	exp Cataract/	16513

25	10 or 11 or 12 or 13 or 14 or 15 or 16 or 17 or 18 or 19 or 20 or 21 or 22 or 23 or 24	208688

26	(test or tests or testing).mp.	1383219

27	examination$.mp.	424043

28	ophthalmoscop$.mp.	9356

29	photoscreen$.mp.	95

30	(acuity or red reflex).mp.	46057

31	exp Vision Tests/or exp Visual Acuity/	52117

32	exp Ophthalmoscopy/	5116

33	(vision or visual).mp.	246181

34	(test$ or screen$).mp.	2011737

35	33 and 34	58712

36	(Hirschberg or Bruckner or motil$ or funduscop$ or cyclopleg$ or skiascop$).mp. [mp = ti, ot, ab, nm, hw]	67108

37	(Auto?refract$ or random?dot or stereoacuity or Snellen or Sheridan-Gardiner).mp. [mp = ti, ot, ab, nm, hw]	2475

38	(Cover?uncover or Alternate cover or Corneal reflex or PhotoScreener or Visiscreen).mp. [mp = ti, ot, ab, nm, hw]	330

39	(Retinomax or Suresight).mp. [mp = ti, ot, ab, nm, hw]	39

40	26 or 27 or 28 or 29 or 30 or 31 or 32 or 35 or 36 or 37 or 38 or 39	1844319

41	exp "Sensitivity and Specificity	211681

42	exp Diagnosis/	3862053

43	diagnos$.mp.	1268941

44	sensitiv$.mp.	743123

45	predict$.mp.	485857

46	accura$.mp.	238100

47	41 or 42 or 43 or 44 or 45 or 46	5133424

48	9 and 25 and 40 and 47	9851

49	exp Contact Lenses/	8117

50	exp Eyeglasses/	4478

51	(refractive adj correct$).ti.	29

52	(refractive adj correct$).ab.	316

53	((optic$ or vision$ or visual$ or filter$ or lens$ or glass$ or spectacle$) adj3 (occlusion or penali$ or patch$)).ti.	129

54	((optic$ or vision$ or visual$ or filter$ or lens$ or glass$ or spectacle$) adj3 (occlusion or penali$ or patch$)).ab.	671

55	49 or 50 or 51 or 52 or 53 or 54	12865

56	exp clinical trials/	190907

57	exp research design/	212573

58	comparative study/or placebos.mp.	1341575

59	exp treatment outcome/	283315

60	double-blind method/or single-blind method/	98869

61	((single or double or triple) adj blind$3).ti.	20568

62	((single or double or triple) adj blind$3).ab.	78020

63	random$.ti.	56160

64	random$.ab.	364411

65	controlled clinical trial.pt.	73779

66	clinical trial.pt.	450604

67	(clinical adj trial$1).ti.	25698

68	(clinical adj trial$1).ab.	90789

69	(control$3 adj trial$1).ti.	15725

70	(control$3 adj trial$1).ab.	38465

71	randomized controlled trial.pt.	228874

72	exp RANDOM ALLOCATION/	57826

73	exp PROSPECTIVE STUDIES/	213531

74	exp Follow-Up Studies/	332322

75	56 or 57 or 58 or 59 or 60 or 61 or 62 or 63 or 64 or 65 or 66 or 67 or 68 or 69 or 70 or 71 or 72 or 73 or 74	2474586

76	9 and 25 and 55 and 75	633

77	9 and 25 and 55	1765

78	screen$.mp.	287728

79	exp Neonatal Screening/	3723

80	exp VISION TESTS/	19699

81	exp MASS SCREENING/	77069

82	78 or 79 or 80 or 81	309225

83	9 and 25 and 82	4644

84	48 or 76 or 83	10737

85	48 or 77 or 83	11540

86	(animals not human).sh.	4025575

87	85 not 86	11140

### Study selection

Titles and abstracts were reviewed using specific inclusion criteria (see below). Full papers of appropriate studies were obtained for detailed evaluation. Authors of studies were contacted if data were unclear or appeared incomplete.

All stages of study selection, data extraction and quality assessment were done independently by two reviewers (CS, RR, SL, RG or JK). Any disagreement during the selection, extraction, and assessment process were resolved by discussion and consensus.

### Inclusion criteria

Included were studies that focused on unselected children from the general population up to the age of six years. Studies which included children with specific diseases (such as diabetes, dyslexia, deafness or congenital diseases) and organic eye defects (such as congenital glaucoma, cataract, retinoblastoma) were excluded. Table 2 shows detailed inclusion criteria.

**Table 2:** Inclusion criteria

**Population**	Children from the general population up to the age of six years
**Intervention**	Comparison of screening versus no screening *or *Comparison of different screening strategies

**Type of study**	Randomised controlled trials Non-randomised intervention studies Controlled cohort studies

**Outcome measurements**	Prevalence rate of amblyopia measured by visual acuity Quality of life (e.g., psychosocial or emotional impairment, labelling, social isolation) Cognitive and educational development Adverse effects related to screening

### Data extraction and quality assessment

For the evaluation of the included studies a modified quality evaluation tool of the Center for Reviews and Dissemination (CRD) was used [26]. Information on the number and age of participants, intervention, sample size planning, blinding of outcome assessor, group comparability, confounding factors, transparency of patient flow, definition of amblyopia and statistical significance of the results was abstracted.

### Statistical analysis

Based on the limitations of the included studies, no meta-analysis or sensitivity-analysis could be performed. Therefore, the results of this review are presented in a narrative way.

## Results

### Results of search and selection process

After removing duplicate references the searches identified 25,944 citations (including potential relevant treatment and diagnostic studies and studies evaluating organic eye diseases). For the question of this review, 24 full text publications (21 studies) evaluating different screening strategies were retrieved for further assessment. Of these, 16 publications (16 studies) were excluded after reading the full paper. Reasons for exclusion and full reference details are given (see Appendix 1).

### Description of included studies

Five studies (eight publications [27-34]) met the a priori defined inclusion criteria. Table 3 shows characteristics and outcome measures of the included studies. The methodological quality of the studies, the prevalence rate of amblyopia and the significance of the results are summarized in Table 4 and 5, respectively.

**Table 3:** Characteristics and outcome measures of the included studies

**Reference**	**Study Type**	**Intervention, number (N) of recruited children and age at screening**	**Number of screened children** **(% coverage)**	**Outcome**
		Orthoptic screening (N = 1582, age: 2.5–3 years)	916 (58)	
		***vs***		
**Bray 1996^33^**	Retrospective cohort study	Health visitor screening (N = 2081, age: 2.5–3 years)	1665 (80)	Prevalence of *amblyopia *at the age of 7 years
		***vs***		
		GP screening (N = 1701, age: 2.5–3 years)	1378 (81)	

		Screening (N = 988, age: 1–2.5 years)	808 (82)	
**Eibschitz-Tsimhoni 2000^27^** **(Israel)**	Retrospective cohort study	***vs***		Prevalence of *amblyopia and visual acuity *in the worse seeing eye at the age of 8 years
		No screening (N = 782)		

		Preschool screening (N = 1347, age 3 years)	1132 (82)	
		***vs***		
**Rasmussen 2000^34^** **(Sweden)**	Randomised controlled clinical trial	No preschool screening (N = 2146)	2097 (98)^§^	Prevalence of *amblyopia and strabismus *at the age of 6.5 years
		*Both groups:* *Current screening programme (age: 4 years)*		

		Intensive screening (N = 2029, age 8,12,18,25,31,37 mos)	1408 (69)	
		***vs***		
**Williams 2002^31,32^** **(UK)**	Pseudo-randomised* controlled clinical trial	Less intensive screening (N = 1461, age 37 mos)	939 (64)	Prevalence of *amblyopia and visual acuity*^† ^in the worse seeing eye at the age of 7.5 years
		*Both groups:* *Current screening programme (age: 48–60 mos)*		

		Preschool screening (N = 1516^‡^, age 37 mos)	1019 (67)	
		***vs***		
**Williams 2003^28,29,30^** **(UK)**	Prospective cohort study	No preschool screening (N = 5062^‡^)	5062	Prevalence of *amblyopia and visual acuity*^† ^in the worse seeing eye at the age of 7.5 years
		*Both groups:* *Current screening programme* *(age: 48–60 mos)*		Psychosocial impairments

* Pseudorandomisation: last digit in day of mother's date of birth was used to assign children to the intervention group.

^† ^Visual acuity of children treated with occlusion.

^‡ ^Only children who took part at the final assessment (at the age of 7.5 years) were presented in the publication.

^§^Refers to the number of children who took part in the current screening programme.

**Table 4:** Methodological quality of the included studies

**Reference**	**Prospective sample size planning**	**Blinding of outcome assessor**	**Comparability of groups**	**Consideration of confounding factors**	**Transparency of patient flow**
**Bray** **1996^33^**	No*	Not specified	Not specified	Not specified	Yes^||^

**Eibschitz-Tsimhoni** **2000^27^**	No	Not specified	Not specified^#^	Not specified	No 82% of the originally recruited children were included in the analysis**

**Rasmussen** **2000^34^**	No	No	Not specified	No	Yes^||^

**Williams** **2002^31,32^**	No^†^	Yes	Yes	Yes^§^	No about 55% of the originally recruited children (equally distributed in both groups) were included in the analysis^††^

**Williams** **2003^28,29,30^**	No*	Yes	No^‡^	Yes^§^	No only children who took part at the final assessment were presented and analysed (62% of the originally recruited children)

^*^ Retrospective power-analysis.

^† ^Retrospective power-analysis for the outcome measure: visual acuity; prospective power-analysis for a not relevant endpoint.

^‡ ^Parents of screened children were older, higher educated and smoked less during pregnancy; however, results were almost identical after adjustment for confounding factors.

^§ ^For example: duration of breastfeeding, mother's educational level, 1st degree relative with squint, sex.

^|| ^No children were excluded from the analysis.

^# ^It was only noted that the two communities were similar in terms of race, social status, health care facilities, education, nutrition and climate.

^**^ Unclear, if only children who attended the final assessment were analysed.

^†† ^Only children who attended the final assessment were analysed.

**Table 5:** Definition and prevalence rate of amblyopia

**Reference**	**Definition of amblyopia** (Snellen acuity)	Prevalence* (Intervention group)	Prevalence* (Control group)	**P-value**
		*Orthoptist*	*Health Visitor*	
	VA ≤6/9	1.1% (0.7–1.8%)	1.0% (0.6–1.5%)	Not reported
**Bray** **1996^33^**			*GP*	
			1.2% (0.8–1.9%)	Not reported

**Eibschitz-**		*Screening*	*No screening*	
**Tsimhoni**	VA ≤5/10	1.0%	2.6%	<0.01
**2000^27^**	VA ≤5/15	0.1%	1.7%	<0.001

**Rasmussen**		*Preschool screening*	*No preschool screening*	
**2000^34^**	VA not specified	0.0%	0.1%	Not reported

**Williams**		*Intensive screening*	*Less intensive screening*	
**2002^31,32^**	Inter-ocular difference ≥ 2 lines	1.5%	2.7%	0.06
	VA <6/12	0.6%	1.8%	0.02

**Williams**		*Preschool screening*	*No preschool screening*	
**2003^28,29,30^**	Inter-ocular difference ≥ 2 lines	1.1%	2.0%	0.05 (0.24)^†^
	VA ≤6/9	1.9%	3.4%	0.01 (0.16)^†^
	VA <6/12	0.7%	1.3%	0.11 (0.55)^†^

VA = Visual acuity.

* In brackets: 95% confidence interval, if reported in the publication.

^† ^Adjustment for confounding factors like sex, highest level of maternal education, birth weight, family history of strabismus/amblyopia and duration of breastfeeding.

Two cohort studies [27,28] and one pseudo-randomised controlled clinical trial [31,32] suggested that screening is significantly associated with an absolute reduction in the prevalence rate of amblyopia between 0.9% and 1.6% (relative reduction: between 45% and 62%). Furthermore, in the retrospective cohort study of Eibschitz-Tsimhoni et al 2000 [27] (the only study which compared screening versus no screening without implementing a current screening programme) it was observed that the frequency of severe amblyopia (visual acuity ≤5/15) was reduced by a factor 17 in the screening group (p < 0.001). Williams 2002 [31,32] and 2003 [28] also reported that mean visual acuity in the worse eye was better for children who had been treated for amblyopia in the intervention group than for similar children in the control group (0.15 versus 0.26 LogMAR p < 0.001; 0.14 versus 0.20 LogMAR p = 0.002, respectively).

However, the reliability of these findings is limited by methodological weaknesses of the studies. For example, Eibschitz-Tsimhoni et al 2000 [27] excluded approximately 20% and Williams 2002 [31,32] approximately 45% of the originally recruited children in their analysis without giving any reasons for exclusion. Williams 2003 [28] only presented children who took part at the final assessment at the age of 7.5 years in their publication. Furthermore, they showed by an "Intention-to-Screen" analysis that the improved outcome for individuals with amblyopia diminished when considering all children offered screening rather than only those who received it. The retrospective cohort study from Bray et al 1996 [33] also found a lack of effects on the prevalence rate of amblyopia at the age of seven years using an "Intention-to-Screen" approach. Despite the fact that orthoptic screening detected more cases of amblyopia associated with microtropia and anisometropia than screening by a health visitor or GP.

The only randomised controlled clinical trial [34] did not find a difference in the prevalence rate of amblyopia between the groups. This study – the only one of the five included studies – also reported a prevalence rate for strabismus at the age of 6.5 years. However, the outcomes were similar in both groups (3.3% [intervention group] versus 3.8% [control group], p = 0.460, Chi^2^-Test, own calculation).

No study conducted prospective sample size planning. Bray et al 1996 [33] and Williams 2002 [31,32], however, showed by retrospective power calculation that the groups had too little power to demonstrate effects [33] or that only moderate effects could be detected [31,32].

The comparability of groups was not given in the cohort study from Williams 2003 [28]. But results were almost identical after adjustment for confounding factors. In the remaining two studies without randomisation [33,27], it was not specified whether factors which could be associated with the main outcome were equally distributed between the groups.

One study [28] evaluated in two additional publications [29,30] the psychological impact (bullying) which patching treatment or wearing glasses might have on children. However, data were not interpretable because of an unclear selection process. Therefore, the relevant question, if such an association depends on the screening model could not be answered. None of the included studies reported data on other patient-relevant outcome parameters.

## Discussion

### Principal findings

Our systematic review identified a lack of rigorous controlled studies examining the effectiveness of a preschool vision screening programme. One of the main limitations of the included studies was that positive effects disappeared when all recruited children were included in the final analysis and not only the sample undergoing screening [28]. This finding confirms that coverage, like compliance with follow-up [35,36], is an important mediator of the effectiveness of a screening programme. For example, in a retrospective cohort study, amblyopia has been found to be as prevalent in screening defaulters as in attenders, indicating that the efficacy of amblyopia detection – and hence the effectiveness of a preschool vision screening programme at all – is highly dependent on its attendance rate [37]. On the other hand, it is possible that the design of the studies made it difficult to find differences between the groups. Only one study compared screening versus no screening [27]. In the remaining studies implemented screening strategies – by means of current screening programmes in the control group – could also have had an effect on the outcome measurement.

Beside methodological limitations, it is important to note that the definition of amblyopia varied across studies. These variations may also effect the outcome measurements. For example, the study of Eibschitz-Tsimhoni 2000 [27] showed that the frequency of moderate amblyopia (visual acuity ≤5/10) was reduced by a factor of 2.5 in children in the screening group. In contrast, screened children with severe amblyopia (visual acuity ≤5/15) showed a prevalence of ambylopia which was reduced by a factor of 17. Bray et al 1996 [33] who used one definition for amblyopia (cut-off visual acuity: 6/9) reported a similar prevalence in all three cohorts. We do not know whether there was a difference between the cohorts in children with more severe amblyopia.

Measures such as school performance, cognitive impairment and quality of life were not adequately evaluated in the reviewed literature. However, concerns about bullying exist [29] and may be a reason to complete treatment (eye patching) prior to school entry.

### Possible damaging effects of preschool screening

Our review has been unable to provide information on the adverse effects of population based preschool vision screening programmes. This is an important omission as concerns about harm exists, particularly from disruption of normal eye development [38], temporary loss of visual acuity in the non-amblyopic eye [39] and costs associated with further evaluation of children with false-positive screening results [40].

The potential psychological impact on the child or its family is also little explored. However, the frequency of these possible damaging effects is primarily dependent on the quality regulations and quality assurance measures in a screening programme.

### Strengths and weaknesses of this review

This review focused on the question of whether preschool screening for amblyopia leads to better vision outcome. Data on diagnostic test accuracy and the effectiveness of interventions will be addressed in separate publications. Extensive effort was invested to identify a wide spectrum of published, unpublished and ongoing studies. We did not apply any language or date restriction. Furthermore, only children from the general population were included as they reflect the screening population. It is most likely that this review contains the most detailed description of the current available literature evaluating preschool vision screening programmes.

### Comparison with other systematic reviews

A Cochrane review from 2005 concluded that insufficient evidence exists to determine the effectiveness of screening programmes on the prevalence of amblyopia [41]. The authors noted that no randomised controlled trials were conducted in this area. An UK assessment from 1997 recommended that screening programmes should not be implemented unless they have been evaluated because there was no evidence found for the benefits of preschool vision screening [22]. A French guideline from 2002 also concluded that a national screening programme for vision disorders cannot be recommended in view of the uncertainties about the power of current screening programmes [42]. In contrast, the USPSTF recommends screening to detect amblyopia, strabismus and other defects in visual acuity in children between three and four years [23]. Similar to our review, the USPSTF found no direct evidence that screening for visual impairment, compared with no screening, leads to improved visual acuity. Their recommendation is based on indirect evidence. For example, they found that treatment of strabismus and amblyopia can improve visual outcomes. In addition, they identified no studies reporting harms resulting from screening, and judged the potential for harms to be small. Therefore, the USPSTF concluded that the benefits of screening are likely to outweigh any potential negative effects. A newly released Canadian Health Technology Assessment from 2007 also concluded that a preschool vision screening programme meets most of the criteria to consider when assessing a screening programme [43]. Still, they added that additional research is needed to ascertain the utility of national preschool vision screening in the Canadian context.

Overall, the cited reviews agree that there is a lack of evidence regarding preschool vision screening. However, the available systematic assessments came to different conclusions. This is most likely due to the fact that different reviews applied different inclusion criteria (for example some reviews also included studies with high-risk children). But it also shows that when reviews are based largely on observational rather than experimental data, their interpretation is likely to be less straightforward.

## Conclusion

The methodological weaknesses of the literature currently available cannot be used to state that preschool vision screening is not effective. But it shows that these programmes have not yet been tested in rigorously controlled trials. Current recommendations should be targeted to maximise coverage in established screening programmes. In future research work screening studies should be developed to compare screened children with children who did not undergo screening (ideally in randomised controlled trials without the implementation of a current screening programme in the control group). However, such a trial might be difficult in particular of ethical reasons. Therefore, different regions with and without screening – for examples in countries like USA, Canada, Belgium, Germany and Switzerland where no standardized preschool vision screening programme is established – should be compared using a controlled study design. Another possibility for such a comparison would be to introduce screening programmes at different time points in different regions (for example three to four year old children should be compared with five to six year old children). The present systematic review also showed that prospective sample size planning should be conducted in such studies. Furthermore bullying and other psychosocial factors should form part of the outcome assessments of screening programmes for amblyopia.

## Abbreviations

USPSTF: U.S. Preventive Services Task Force

## Competing interests

The authors declare that they have no competing interests.

## Authors' contributions

JK, RG and SL developed the protocol and design of the study. Study selection and data extraction was carried out by CS, JK, SL, RG and RR. WL provided clinical advice and GA provided methodological support. All authors were involved in data interpretation and had full access to all of the data. CS wrote the paper. All authors commented on drafts of the paper and approved the final version.

## Appendix 1

References of excluded studies and reasons for exclusion after reading the full paper

Atkinson J, Braddick O: **Population vision screening and individual visual assessment**. *Doc Ophthalmol Proc Series *1986, **45**:376.

*Reason for exclusion: no relevant outcome measurements*.

Donahue SP, Baker JD, Scott WE, Rychwalski P, Neely DE, Tong P, et al: **Lions Clubs International Foundation Core For Photoscreening: results from 17 programs and 400,000 preschool children**. *Journal of AAPOS *2006, **10**:44–8.

*Reason for exclusion: no relevant outcome measurements*.

Hard AL: **Results of vision screening of 6-year-olds at school: a population-based study with emphasis on screening limits**. *Acta Ophthalmol Scand *2007; **85**:415–8.

*Reason for exclusion: no comparison of different screening strategies*.

Hard AL, Sjodell L, Borres MP, Zetterberg I, Sjostrand J. **Preschool vision screening in a Swedish city region: results after alteration of criteria for referral to eye clinics**. *Acta Ophthalmol Scand *2002, **80**:608–11.

*Reason for exclusion: no relevant outcome measurements*.

Jarvis SN, Tamhne RC, Thompson L, Francis PM, Anderson J, Colver AF: **Preschool vision screening**. *Arch Dis Child *1991, **66**:288–94.

*Reason for exclusion: no relevant outcome measurements*.

Juttmann RE, Van der Maas PJ, Lantau VK, Simonsz HJ, De Faber JTN, Van der Werf-De Koning CM, et al: **The Rotterdam Amblyopia Screening Effectiveness Study (RAMSES): Compliance and predictive value in the first 2 years**. *Br J Ophthalmol *2001, **85**:1332–5. *Reason for exclusion: no comparison of different screening strategies*.

Kemper AR, Clark SJ: ***Preschool Vision Screening by Family Physicians***. *J Pediatr Ophthalmol Strabismus *2007, **44**:24–7.

*Reason for exclusion: no comparison of different screening strategies*.

Kemper AR, Uren RL, Clark SJ: **Barriers to Follow-up Eye Care After Preschool Vision Screening in the Primary Care Setting: Findings From a Pilot Study**. *Journal of AAPOS *2006, **10**: 476–8.

*Reason for exclusion: study type not eligible*.

Kohler L, Stigmar G: **Vision screening of four-year-old children**. *Acta Paediatr Scand *1973, **62**:17–27.

*Reason for exclusion: no comparison of different screening strategies*.

Kvarnström G, Jakobsson P, Lennerstrand G: **Screening for visual and ocular disorders in children of the system in Sweden**. *Acta Paediatr *1998, **87**:1173–79.

*Reason for exclusion: no comparison of different screening strategies*.

Macchiaverni FN, Jose NK, Rueda G: **Ophthalmologic screening of schoolchildren in Paulinia, Sao Paulo (Brazil)**. *Arq Bras Oftalmol *1979, **42**: 289–94.

*Reason for exclusion: selected group of children*.

Morad Y, Bakshi E, Levin A, Binyamini OG, Zadok D, Avni I, Dayan YB: **Screening and Treating Amblyopia: Are We Making a Difference? ***Invest Ophthalmol Vis Sci *2007, **48**:2084–88.

*Reason for exclusion: no comparison of different screening strategies*.

Nelson LB: **Preschool vision screening by family physicians**. *J Pediatr Ophthalmol Strabismus *2007, **44**:12.

*Reason for exclusion: study type not eligible*.

Pampapathi MR, Cadman A: **Screening for squints and amblyopia in pre-school children in a service community**. *J R Army Med Corps *1990, **136**:153–5.

*Reason for exclusion: no relevant outcome measurements*.

Schaeffel F, Mathis U, Bruggemann G: **Noncycloplegic photorefractive screening in pre-school children with the "PowerRefractor" in a pediatric practice**. *Optom Vis Sci *2007, **84**:630–9.

*Reason for exclusion: no comparison of different screening strategies*.

Zaba JN, Reynolds W, Mozlin R, Costich J, Slavova S, Steele GT: **Comparing the effectiveness of vision screenings as part of the school entrance physical examination to comprehensive vision examinations in children ages 3 to 6: an exploratory study**. *Optometry *2007, **10**:514–22.

*Reason for exclusion: selected group of children*.

## Pre-publication history

The pre-publication history for this paper can be accessed here:


